# Predicting the theory of mind precursors based on parenting styles and language skills in preschool children

**DOI:** 10.22037/ijcn.v15i2.23235

**Published:** 2021

**Authors:** Hamid KHANIPOUR, Sara NEZAFATBAKHSH, Naser AGHABABAEI, Samira ZAND

**Affiliations:** 1 Institute of Educational, Psychological, and Social Research, Kharazmi University, Tehran, Iran.; 2 Islamic Azad University - E-Campus (IAUEC), Tehran, Iran; 3 Department of Behavioral Sciences, Institute for Research, and Development in the Humanities (SAMT), Tehran, Iran; 4 Islamic Azad University, Zanjan Branch, Zanjan, Iran.

**Keywords:** Theory of mind, Language, Parenting, Preschool children

## Abstract

**Objective:**

The current study aimed to investigate the association between language skills and parenting styles and three levels of theory of mind (including emotion recognition, false belief understanding, and second-order belief) among Iranian preschool children.

**Materials & Methods:**

A total of 98 preschool Iranian children (aged 5-6 years) living in the Karaj province, Iran were recruited. While the theory of mind test and test of language development (Told-p: 4) were administrated on children, their mothers were asked to answer a parenting style questionnaire.

**Results:**

Multivariate regression analysis showed a differential association between three levels of theory of mind, language skills, and parenting styles. Most language skills and permissiveness parenting styles could predict the emotion recognition ability (P<0.01). Morphological completion predicted false belief understanding (p<0.01). Also, word articulation and authoritative parenting style could predict the ability to understand second-order belief (R^2^=28%).

**Conclusion:**

The pattern of associations between language and theory of mind in the Persian language seems similar to previous studies in other languages. Language may play a dual role in the theory of mind. Whereas for the first (basic) and the third (advanced) level of theory of mind, language skills, like relational vocabulary, morphological completion, and word articulation, are general and nonspecific predictors, but syntactical skills are a specific casual predictor for the false belief understanding. Also, it seems that an authoritative parenting style could facilitate the development of higher-order abilities related to the theory of mind.

## Introduction

Theory of mind (TOM) is a major skill in social interactions. It refers to the ability for identifying, predicting, and controlling other people's subjective experiences (1). TOM has been linked to children’s social and moral development (3) and was conceptualized at three levels (4). The first level deals with the ability to understand and to differentiate the emotional states of other people. In the second level, TOM addresses cognitive processes involved in understanding false beliefs or the ability to ascribe different beliefs to people. Finally, the third level is about understanding irony, metaphor, and allegory (4, 5). 

According to the Modularity theory (6, 7), cognitive processes of TOM and general cognitive skills, such as problem-solving and language skills, are different skills. However, some researchers argued about the interactional nature of skills involved in the TOM (8, 9), such that biological, environmental, and cultural factors contribute to the development of TOM (1). Hence, it can be argued that the ability to succeed in tests related to TOM depends on the development of cognitive skills such as language (10, 11) and parenting styles (12, 13). Previous studies showed that each of these factors acts as the origin of individual differences in TOM, but the strength of the association between each of these predictors and three different levels of TOM cannot be fully identified. These two factors were selected because of controversies regarding their role as predictors of TOM (14) and the fact that both of them may be influenced by culture. As a result, investigating these two factors can provide useful information regarding the universality of the association between language, parenting styles, and TOM.

Three essential language skills are thought to be semantical knowledge, syntax, and pragmatics (15). Nevertheless, there are controversies regarding the association between TOM and language skills. Meanwhile, language skills, as prerequisites for success in verbal measures of tests related to the TOM, have been underscored (16, 17). On the other hand, specific language skills, such as syntax processing, are believed to have an important role in developing TOM (10, 18). Syntax processing refers to syntactical understanding and complementation, which are associated with the ability to both understand and apply the grammatical principles (15). Previous studies showed that learning the mental state words and ability to use syntactical rules to construct complex sentences with multiple propositions (or the grammatical ability to complement the main clause with a subordinate clause in a sentence), named as syntactical complementation, could longitudinally predict false belief understanding among preschool children (19, 20, 21), but it is not clear whether these linguistic abilities can predict other levels of TOM or not. 

Some studies about the role of language on the development of TOM advocated the idea of TOM deficiency in children with language disorders. A meta-analysis study revealed that children with specific language impairments had lower TOM performance compared to typically developed children (11). Evidence regarding the delayed development of TOM in children with specific language impairments (22) corroborated the importance of language in predicting TOM. One of the main debates in literature has been whether there is a specific association between language and social cognition or they are general interrelated cognitive skills (1). A recent longitudinal study on the contribution of general verbal abilities suggested that the ability of two and half years old children in using mental state words like “think” and “belief” could predict the performance in TOM tasks beyond general verbal ability (18). There is a general consensus that language skills can predict TOM (17, 18, and 19). However, there has not yet been a closer picture of this association.

Although most of the typically developed five-year-old children could successfully pass the TOM tasks (1), there are considerable variations or individual differences in children’s performances in TOM tasks. It is believed that several factors affect the TOM, including demographic, environmental, and social factors (9). For instance, parenting style is reported as a social factor that contributes to the development of TOM (8), which entails parents’ methods to rear their children as well as their responses to the emotional needs of children (23). Three types of parenting styles are authoritarian, authoritative, and permissive (23). Some studies partially provided evidence regarding the role of parents’ communication types on TOM development (12, 13). Previous studies revealed that parental power-assertive practices, which were characterized by an optimal balance of emotional support and encouraging autonomy in child-rearing, were negatively associated with a low score in belief understanding tasks (9). Despite the commonsense belief that authoritative parenting style may aid children to figure others’ perspectives more easily, no study has yet concluded a relation with later success in TOM tasks by children. Nevertheless, some studies reported a positive association between power assertive parenting practice and emotional understanding in children (24, 25). 

Knowledge about the association between TOM, language, and parenting styles might hold implications with respect to educational methods or rehabilitation programs for children in general. One of the distinguishing advantages of the current study is the focus on three different levels of theory of mind. Previous studies have solely focused on the antecedents of false belief understanding. Another advantage of this study is proposing the idea of differential contributions of language skills and parenting styles on the development of TOM. Thus, the current study aimed to investigate possible associations between language skills, parenting styles, and three levels of TOM. We hypothesized that language and parenting styles can differentially predict levels of TOM. It worth noting that according to the best knowledge of the authors, no study has focused on the associations between TOM, language skills, and parenting in Iranian children. Therefore, the current study aimed to investigate the differential role of three kinds of parenting styles and language skills, as precursors of TOM, among preschool children.

## Material & Methods

Following a cross-sectional design, 98 preschool children (aged 5 to 7 years) and their mothers, who at the time of performing the study were living in Karaj Province (Iran), were recruited in this correlational research. The Inclusion criteria were (1) being aged 5 to 7 years, (2) being monolingual, (3) no history of emotional or behavioral disorders, and (4) no speech or language development disorder like stuttering. Participants were selected using the convenient sampling technique. A total of 98 children (37 girls and 61 boys) and their mothers participated in the present study. Data on academic degree and job status of parents are provided in Table 1. Among families who enrolled in this study, 69 (70%) had only 1 child, and 29 (30%) families had at least two children. 

**Table1:** Frequency of parent’s academic degrees and their job status

		Father	mother
Academic degree	High school diploma	24(24.5%)	27(27.6%)
	Bachelor	69(70.4%)	62(63.3%)
	Master	5(5.1%)	9(9.2%)
Job	Householder	0	67(68/4%)
	Teacher	24(24.5%)	0
	Employees	16(16.3%)	22(22.4%)
	Self-employed	58(59.2%)	9(9.2%)

Participants were selected from four preschool education centers in Karaj province. Initially, we invited mothers to participate in a meeting in order to ask them to participate in the present study. Those who were willing to participate, along with their child/children, attended another meeting with researchers of the study. In this meeting, all necessary information about the objectives of the study and its procedures were provided to them. They were then asked to fill in the parenting style questionnaire. In the following, by getting accustomed to the testing environment and creating a feeling of intimacy between researchers and children, tests related to the TOM and language development were administered. To thank participating mothers, a series of pamphlets on cognitive skills in children and education strategies were given to them. The whole session, on average, lasted for one and a half hours. Informed written consent was obtained from all participating mothers. In addition, we obtained their permission to administer TOM and language-related tasks to their children. The study is approved by the State Welfare Organization of Iran, which administers the issues related to kindergartens in Iran. A local child clinical psychologist examined all children individually at the preschool. 

**Instruments**

1-**Theory of Mind Test (TOM test)**

In order to assess TOM, a short form of the test with 38 items (5, 26) was applied. This instrument is developed to investigate multiple levels of TOM. The original test entails several items for various scenario-based situations to which children are asked to answer. Recognition of emotions, false belief understanding, and second-order belief were regarded as three subscales of the test (5). The internal consistency of this instrument on a sample of Iranians was evaluated by Cronbach alpha, which for each of the three subscales, it was 0.86, 0.72, and 0.80, respectively (27).

2- **Parenting authority questionnaire (PAQ)**

Parenting style was evaluated using PAQ, which is designed to evaluate three main styles of parenting (28). It contains three main subscales measuring authoritarian, permissive, and authoritative styles. It consists of 30 items scored on a 5-point Likert scale with relatively high validity and reliability (29). This questionnaire has been adapted using a sample of Iranians, and the internal consistency of subscales is calculated as 0.69, 0.77, and 0.73 (30). The mothers were asked to individually fill the questionnaire based on their own practiced parenting style.

3- **Test of language development (Told-p: 4)**

This test was used to monitor the development of various skills related to language (31). It has nine subscales of picture vocabulary, relational vocabulary, oral vocabulary, syntactic understanding, sentence imitation, morphological completion, word discrimination, word analysis, and word articulation (31). The test was normalized in a sample of Iranian children (32). The factor analysis revealed a one-factor solution for nine subscales, which advocated consistency with the underlying theory of the test (32). We used the Persian version of Told (32), which has a similar factors structure among Iranian preschool children, as the original English version. We administered four subscales of relational vocabulary, oral vocabulary, syntactic understanding, morphological completion, and word articulation. In the following, subscales that were more related to semantical and syntactical aspects of language were selected. Items related to semantic processing are intended to evaluate the lexicon knowledge, and items regarding syntax processing are designed to examine the ability to understanding and apply syntactical rules of language. 

## Results

The mean age of girls and boys was 6.1 and 6.4 years, respectively. There were significant differences concerning some sub-scales of TOM and language skills among children (i.e., girls obtained higher scores).

**Table 2:** Comparison of the theory of mind levels and language skills, separated by gender

		Girls M(sd)	Boy M(sd)	wilk's lambda	F	sig
*Theory of mind*	TOM 1	17.2(2.1)	15.7(2.3)	3.5	10.66	0.002
	TOM 2	10.5(1.8)	9.82(1.87)		3.22	0.07
	TOM 3	1.7(1.5)	1.36(1.6)		0.966	0.328
*Language ability*	Relational vocabulary	17.8 (4.9)	13.6 (4.4)	4.50	16.8	0.001
	Oral vocabulary	14.9 (4.8)	13.9 (4.9)		1.14	0.287
	syntactic understanding	15.2 (4.2)	12.7 (4)		8.67	0.004
	Morphological completion	14.8 (4.9)	11.6 (4.7)		10.06	0.002
	Word articulation	15 (3.7)	13.8 (3.2)		2.8	0.094

As shown in Table 2, according to the results of multivariate analysis of variance, in the first level of TOM (i.e. emotion recognition), girls obtained higher scores than boys (F=10.66, *p*=0.002). As shown in Table 2, for other levels of TOM, there was no significant difference. In addition, there were significant differences concerning relational vocabulary (F=16.8, *p*=0.001), syntactic understanding (F=8.67, *p*=0.004), and morphological completion (F=10.06, *p*=0.002) between girls and boys. For all variables, girls had higher scores than boys. 

Correlation and multivariate regression analyses were conducted to investigate the association between parenting styles, language skills, and three levels of TOM. The zero-order correlation is provided in Table 3. There were significant associations between each level of TOM, parenting styles, and language skills.

**Table 3:** Correlations between TOM levels, language skills, and parenting styles

	*TOM1*	*TOM2*	*TOM3*	*RV*	*OV*	*SU*	*MC*	*WA*	*Permissiveness*	*authoritarian*	*authoritative*
*TOM1*	1	0.59**	0.38*	0.73**	0.55**	0.62**	0.68**	0.45**	0.48**	-0.36**	0.48**
*TOM2*	0.59	1	0.27	0.42**	0.33**	0.29**	0.52**	0.25**	0.22*	-0.20*	0.30*
*TOM3*	0.38	0.27	1	0.18*	0.33**	0.20*	0.23*	0.44**	0.13*	-0.18*	0.29*
*RV *	0.73	0.42	0.18	1	0.45**	0.45**	0.59**	0.31**	0.28**	-0.39**	0.44**
*OV*	0.55	0.33	0.32	0.45	1	0.51**	0.43**	0.35**	0.27**	-0.17**	0.27*
*SU*	0.62	0.29	0.20	0.45	0.51	1	0.43**	0.22*	0.33**	-0.22*	0.38*
*MC*	0.68	0.52	0.23	0.59	0.438	0.43	1	0.31**	0.38**	-0.42**	0.45**
*WA*	0.45	0.25	0.44	0.31	0.35	0.22	0.31	1	0.17*	-0.18*	0.13
*Permissiveness*	0.48	0.22	0.13	0.28	0.27	0.33	0.38	0.17	1	-0.37**	0.24*
*authoritarian *	-0.36	-0.27	-0.18	-0.39	-0.17	-0.22	-0.41	-0.18	-0.37	1	-0.39
*Authoritative*	0.48	0.30	0.29	0.44	0.27	0.38	0.45	0.13	0.24	-0.39	1

Note: (RV: Relational vocabulary, OV: oral vocabulary, SU: syntactic understanding, MC: Morphological completion, WA: Word articulation)

*P< 0.05, ** p< 0. 001

The results of multivariate regression are provided in Table 4. As shown in the Table, relational vocabulary, syntactic understanding, morphological completion, and word articulation could predict the first level of TOM. This model could predict 74% of the variance. 

**Table 4:** The results of multivariate regression analysis

*Dependent variables*	Predictor variables	*B*	*Sd*	*t*	*sig*
*TOM 1 *	RV	0.161	1.31	4.36	0.001
	OV	0.034	0.035	0.992	0.324
	SU	0.122	0.037	3.310	0.001
	MC	0.106	0.034	3.123	0.002
	WA	0.118	0.039	3.01	0.003
	Permissiveness	0.057	0.019	3.001	0.003
	Authoritarian	0.020	0.023	0.854	0.395
	Authoritative	0.021	0.019	1.14	0.256
*TOM 2 *	RV	0.051	0.043	1.177	0.242
	OV	0.026	0.047	0.564	0.574
	SU	0.010	0.050	-0.017	0.986
	MC	0.140	0.046	3.073	0.003
	WA	0.039	0.053	0.736	0.464
	Permissiveness	0.006	0.025	0.541	0.590
	Authoritarian	0.018	0.031	0.584	0.561
	Authoritative	0.014	0.025	0.541	0.590
*TOM 3 *	RV	-0.045	0.039	-1.16	0.249
	OV	0.075	0.042	1.775	0.874
	SU	-0.007	0.045	-.159	0.874
	MC	-0.005	0.041	-0.110	0.913
	WA	0.184	0.048	3.86	0.001
	Permissiveness	-0.003	0.023	-.135	0.893
	Authoritarian	-0.016	0.028	-.553	0.582
	Authoritative	0.051	0.023	2.21	0.029

Note: (RV: Relational vocabulary, OV: oral vocabulary, SU: syntactic understanding, MC: Morphological completion, WA: Word articulation)

As shown in Table 4, the beta coefficients of variables showed that the total effect of each of language skills was higher than parenting styles. For the second level of TOM, analysis of the predictive role of variables indicated that only morphological completion could predict 30% of the variance of false belief understanding tasks. Finally, for the third level of TOM, regression analysis revealed that word articulation and authoritative parenting style could predict the third level of TOM (R^2^=28%).

## Discussion

The current study aimed to investigate the role of language skills and parenting styles in the prediction of TOM. The findings indicated a specific pattern of association between language skills and parenting styles across different levels of TOM. The first level of TOM related to the importance of skills such as emotion recognition, which were considered as the precursor for the development of real TOM (4). Language skills, including relational vocabulary, syntax understanding, morphological completion, word articulation together with permissive parenting style, could predict the first level of TOM. Hence, it can be argued that first-level tasks of TOM may require more general cognitive abilities such as language. Thus, it can be inferred that these language skills acted as precursors of real TOM; as there was a strong association between language skills and TOM. In other words, language skills contributed to integrative cognitive ability for the development of the first level of TOM. Only permissive parenting styles were associated with the first level of TOM, which is inconsistent with previous findings that support the idea of the positive role of authoritative parenting in facilitating children’s empathy with victim’s emotions (33). This difference may show that the first level of TOM (i.e. emotion recognition) depends on the child him/herself, rather than parenting styles. It seems that the first level of TOM is more related to the ability to distinguish basic emotions, as previous facial expression studies (34) revealed that people in every culture can distinguish these universal emotional signs. In the present study, the subtests of the first level of TOM intended to evaluate the ability to understand other people’s emotions and feelings. Noteworthy, such an ability is supposed to be innate or has a universal nature (34). The findings of the present study indicated that acquisition of basic aspects of TOM (i.e., emotion recognition) might depend on inner developmental processes rather than parenting styles. In other words, it can be suggested that every typical child in any culture has the biological or evolutionary preparedness to distinguish and recognize basic emotions. Nevertheless, it is not true for all emotions, particularly regarding complex emotions or self-conscious emotions like shame, guilt, and pride that are more sensitive to the developmental experiences, including parenting styles, and require socialization through parenting styles (23, 34). Future studies should focus on the differential role of parenting styles and language skills on the development of recognition or expression of complex emotions.

Furthermore, only morphological completion, which included all variables of language skills as well as parenting styles, could predict the second level of TOM or false belief understanding. This finding is consistent with previous studies, which indicated that grammatical knowledge in word-level was more related to the false belief understanding compared to other language skills (e.g., semantic and pragmatics in children with either typical or atypical development) (11, 18, 21, 36). It seems that the ability to represent other people’s minds requires applying syntactical completion rules. Such a specific association between syntax and the second level of TOM has been evidenced by suggesting the predictive role of morphological completion ability and the development of false belief understanding (19, 20, 21). In other words, to represent and perceive other people’s beliefs, it is necessary to understand and apply language grammar, which in turn allows children to construct complex sentences with multiple propositions. There are controversies regarding the association between language skills and false belief understanding. While some studies supported the association of syntactical ability with false belief understanding (11, 18, 21, 36), there were also studies that indicated no language-specific association between language and false belief understanding (37). The findings of the present study supported the role of morphological completion in better predicting false belief understanding and moving beyond general language skills and parenting styles. In addition, the observed non-significant association between parenting styles and the second level of TOM (i.e., false belief understanding) is in line with the modularity nature of TOM (6, 7). In other words, false belief understanding is more a specific cognitive ability and is developed independently from environmental factors and general cognitive skills (1, 6, and 7). 

Moreover, the third level of TOM (i.e., the second-order belief) was predicted by the skill to articulate words and authoritative parenting style. As mentioned earlier, this level deals with understanding irony, humor, and belief about beliefs. In order to examine items related to word articulation, phonology and semantic knowledge were investigated. This finding is consistent with the currently available evidence, which does not support language-specific skills as predictors of second-order TOM (35, 37). However, our finding indicated that other aspects of TOM were strongly associated with language skills. 

It is well-proved that the general verbal ability is correlated with the ability to succeed in performing the TOM tasks (10, 38, 39). Nevertheless, the findings of the present study suggested a specific association between language and TOM. In sum, there were differential associations between language skills and three levels of TOM. In contrast, syntax was specifically correlated with the typical task of TOM (i.e. false belief understanding task). In addition, all language skills were related to precursors of TOM (i.e. emotion recognition). Moreover, in the present study, there was an overall association between language and the third level of TOM (i.e. second-order belief); in that, only general ability to articulate words was related to this level of TOM. In other words, it seems that language might act as a highly general and nonspecific prerequisite ability for the first and third levels of TOM development, especially when it comes to understanding second-order beliefs and emotion recognition. Whereas language, particularly syntax, may be a casual prerequisite for performing false belief tasks (i.e., the second level of TOM).

Moreover, the findings showed that authoritative parenting style could predict the third level of TOM, which is consistent with the findings of a previous study that indicated parents who were encouraging their children to understand other people’s feelings in every interaction could facilitate the development of TOM of their children (13). It can be argued that parents who follow an authoritative parenting style talk more about mental states like desire, opinion, and other people’s emotions, and this kind of verbal elaboration is associated with better mentalization process in their children, which in turn their children present a better performance in TOM tasks. Furthermore, compared to the first level, the third level of TOM was more strongly influenced by parenting style. 

Concerning the item about specialty or generality of the relationship between language and TOM, our findings are consistent with previous studies (11, 18, 21, 36), which supported the specific role of syntax in performing explicit tasks of TOM. Nevertheless, we found that all language skills are necessary for achieving TOM tasks. In addition, the ability to perform higher orders of TOM was less affected by language skills, except for word articulation. Noteworthy, the results revealed similar patterns of association between language and TOM for Farsi language, as consistent with previous studies (11). 

As we followed a cross-sectional design, the generalizability of the results to different contexts is restricted, as it was not possible to investigate the directions of effects or causalities. Future studies should follow a longitudinal design that allows to closely investigate the causal effects of diverse social and linguistic variables on TOM. Also, future studies should examine the interaction between language and parenting styles and should focus on how this interaction may affect performance in the theory of mind tasks.

## In Conclusion

This study demonstrated that linguistic ability and parenting styles are associated with individual differences in TOM. According to the findings, it can be suggested that facilitating syntactical ability may be an effective strategy to overcome possible defects or lags in TOM among children. Furthermore, based on the association between authoritative parenting styles and second-order TOM, it can be concluded that the more parents apply democratic rules and the more they foster independence in their children, the more competency would emerge in the abilities related to second-order TOM, like understanding irony, mentalization, and logical or critical thinking. 
